# Genetic Rescue: Latest Advances and Applications

**DOI:** 10.1111/eva.70225

**Published:** 2026-03-19

**Authors:** Luciano B. Beheregaray, Sarah W. Fitzpatrick, Andrew R. Whiteley, Jill Anderson, Paul Sunnucks

**Affiliations:** ^1^ Molecular Ecology Laboratory, College of Science and Engineering Flinders University Bedford Park South Australia Australia; ^2^ W.K. Kellogg Biological Station, Department of Integrative Biology Michigan State University East Lansing Michigan USA; ^3^ Wildlife Biology Program, Franke College of Forestry and Conservation University of Montana Missoula Montana USA; ^4^ Department of Genetics and Odum School of Ecology University of Georgia Athens Georgia USA; ^5^ Wildlife Genetic Management Group, School of Biological Sciences Monash University Clayton Australia

**Keywords:** conservation genetics, conservation management, endangered species, genetic load, genomic erosion, inbreeding depression

## Abstract

Genetic rescue is the increase in individual or population fitness caused by new genetic variation. It typically involves the deliberate movement of genetically diverse individuals into small and isolated populations to reduce inbreeding and maladaptation while enhancing evolutionary potential. Despite growing interest, gaps in theory and empirical evidence have limited the wider application of genetic rescue in conservation biology. To address these gaps, we put together this Special Issue on Genetic Rescue. The issue assembles 21 original papers that evaluate or apply genetic rescue and related approaches in conservation. The contributions include empirical studies and simulations spanning diverse animal and plant species across varied ecological and socio‐environmental contexts, alongside perspectives and syntheses. Collectively, they demonstrate the value of integrating population genomics and evolutionary biology into conservation, identify opportunities and limitations of genetic rescue, and underscore its potential to improve resilience, adaptive capacity, and the long‐term persistence of biodiversity.

## 1 A Special Issue on Genetic Rescue

Rapid, human‐induced global change presents an urgent challenge for conservation and evolutionary biologists, natural resource managers, and the broader public (Thomas et al. 2004; Urban 2024). The ongoing deterioration, fragmentation and loss of habitat drives population declines, reduces genetic variation, increases inbreeding, disrupts connectivity, and imposes selection pressures and cascading community effects that accelerate the biodiversity extinction crisis (Reed 2004; Brook et al. 2008; Haddad et al. 2015; Shaw et al. 2025). To counter these trends, management strategies should aim to maintain metapopulations that are large, genetically diverse, naturally connected, and distributed across a wide range of environmental conditions (Haddad et al. 2015; Webster et al. 2017). Building evolutionary resilience into populations further requires incorporating evolutionary processes into conservation, landscape, and aquascape planning (Sgrò et al. 2011; Grummer et al. 2019). This includes preserving or restoring, wherever possible, the genetic variation within populations and species—both neutral and adaptive genetic variation (García‐Dorado and Caballero 2021). Genome‐wide genetic variation is positively associated with reproductive success, viability, growth rate, gamete quality, and disease resistance (reviewed in DeWoody et al. 2021). In line with this, genome‐wide heterozygosity, quantitative genetic variation, and population size are all positively linked to population fitness (Reed and Frankham 2003), highlighting how the erosion of genetic variation undermines long‐term viability (Kardos et al. 2021).

**FIGURE 1:**
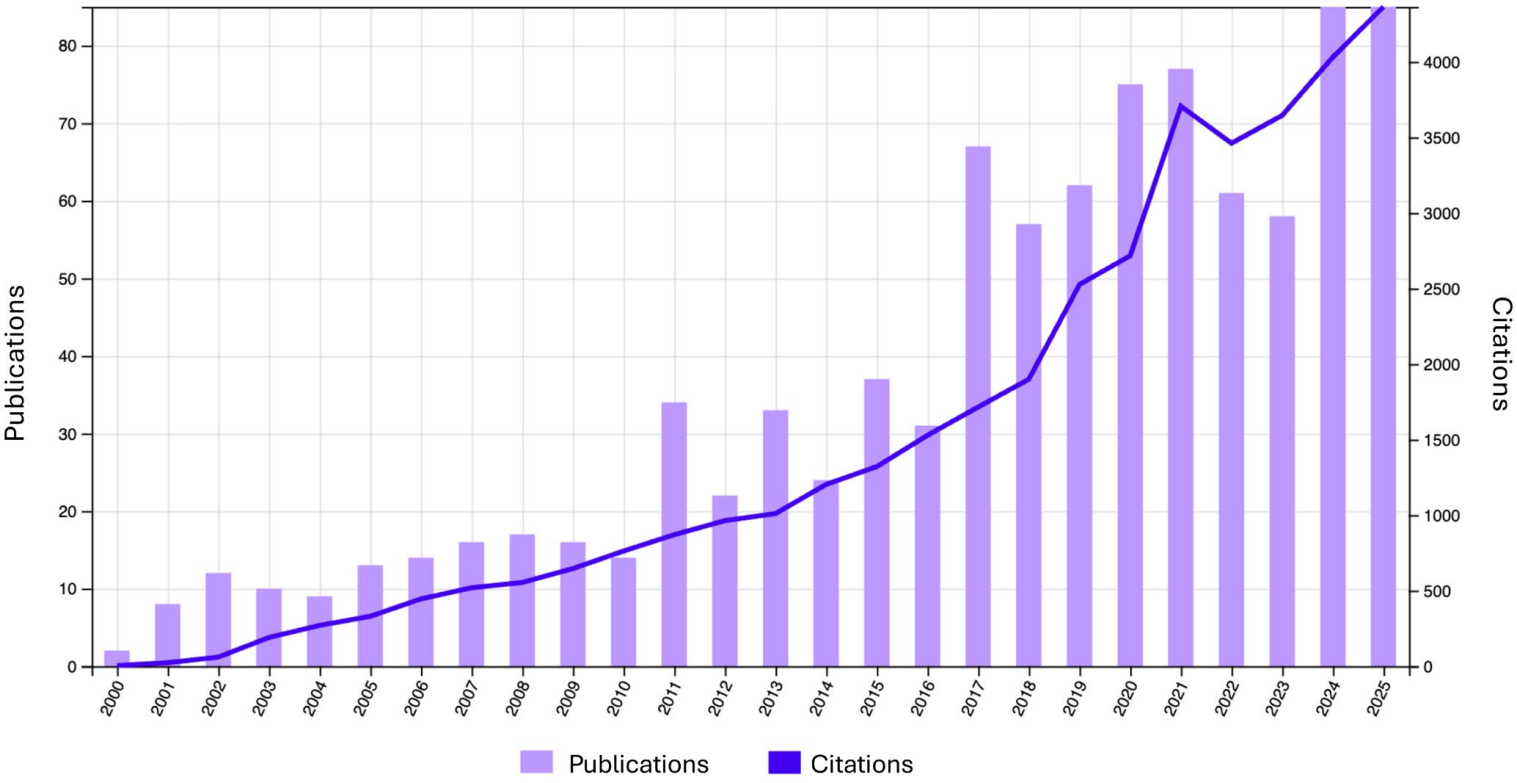
Number of articles mentioning “genetic rescue” (2000–2025) and the citations they have received. Data retrieved on 30 December 2025 from Clarivate Web of Science.

An increasingly recognised outcome of managed gene flow in conservation biology is *genetic rescue* (Figure 1). Managing for genetic rescue typically involves deliberately moving individuals from a genetically diverse population into a threatened one to reduce inbreeding, boost population growth, and enhance the target population's genetic diversity and evolutionary potential (Frankham 2015; Whiteley et al. 2015). Genetic rescue and related strategies, such as assisted gene flow (e.g., Aitken and Whitlock 2013), involve human‐assisted migration to (re)introduce new allelic variation into small and declining populations. This can reduce maladaptation associated with realised genetic load (the expression of deleterious alleles) and lead to fitness gains that exceed those attributable to the demographic contribution of immigrants alone (Tallmon et al. 2004; Whiteley et al. 2015; Fitzpatrick et al. 2016). An increase in population growth rate is often considered a key indicator of genetic rescue success; however, this approach may also be appropriate for populations that are currently demographically abundant but still suffer from genetic health issues resulting from past severe bottlenecks (e.g., Gates et al. 2025). Genetic rescue can be a powerful goal when managing threatened populations, especially when combined with other measures that address non‐genetic drivers of decline, such as habitat restoration, life‐history attributes, and the control of predators, competitors, and invasive species (Tallmon et al. 2004; Bell et al. 2019; Weeks et al. 2017), particularly when accompanied by best‐practice monitoring to evaluate genetic rescue efforts (Robinson et al. 2021).

Although assessing and forecasting the fitness consequences of outcrossing wild populations is inherently complex, theory and empirical evidence clearly indicate that gene flow can be beneficial for small, isolated, and inbred populations (Slatkin 1985; Frankham 2015). Nonetheless, concerns that gene flow may lead to outbreeding depression or introduce harmful variation have created uncertainty and limited the broader adoption of genetic rescue as a management strategy (Fitzpatrick et al. 2023). Such concerns are typically context‐specific and can be exaggerated or based on unrealistic modelling scenarios (Ralls et al. 2020; Willi et al. 2022). In addition, evidence from multiple species indicates that inbreeding depression occurs more often and is generally more severe than outbreeding depression (Frankham et al. 2017; Liddell et al. 2021). Meta‐analyses (e.g., Frankham 2015), together with numerous recent theoretical and empirical studies (examples in this Special Issue), demonstrate that introducing outbred individuals to maximise genetic diversity in the target population remains the prevailing recommendation for genetic rescue (Whiteley et al. 2015; Ralls et al. 2020; Clarke et al. 2024). Guidelines and decision‐support tools for planning and implementing genetic rescue have been proposed (Hedrick and Fredrickson 2010; Frankham et al. 2011, 2017; Tengstedt et al. 2026). However, despite the increasing availability of genetic data, conservation interventions and decisions about whether to mix gene pools often lack a genetic risk assessment (Liddell et al. 2021). Evaluations of conservation actions using experimental tests of fitness gains (Pavlova et al. 2023; Wilder et al. 2025), individual‐based simulations (Robinson et al. 2021), and field monitoring (Marshall et al. 2022) are expected to reduce uncertainty and encourage the use of genetic rescue.

**FIGURE 2:**
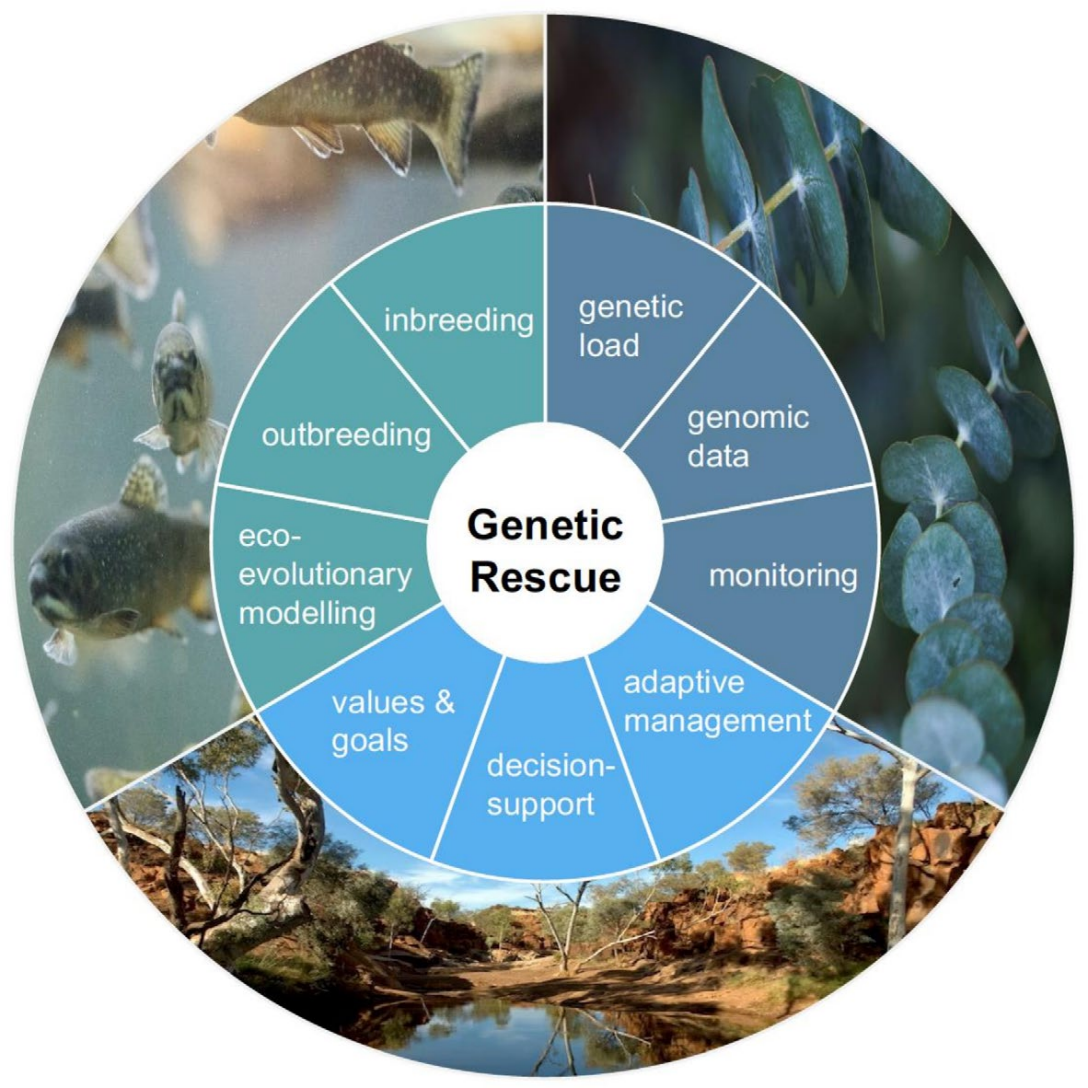
The topics addressed in the 21 articles published in this Special Issue on Genetic Rescue include empirical, modelling, and theoretical approaches.

This Special Issue on Genetic Rescue highlights substantial advances made in recent years in the use of population genomics for the management of threatened species. It includes a broad range of studies that apply or assess the value of genetic rescue and related concepts, such as evolutionary rescue, genetic restoration, or genetic mixing, for conservation purposes (Figure 2). Empirical applications examined wild and/or captive populations of birds, carnivores, ungulates, rodents, marsupials, teleosts, butterflies, gastropods, crop pest–predator systems, grassland plants, and legumes. Perspectives, reviews, syntheses, and modelling studies explored issues such as spatial and environmental structure under habitat loss, mitonuclear replacement, and demo‐genetic simulations of genetic rescue.

## 2 Integration of Genomic, Simulated, Field‐ and Captive‐Based Datasets

### 2.1 Planning for Genetic Rescue and Assessing Outcomes

A limitation preventing progress in conservation genetics relates to the lack of empirical and simulated data to inform on suitable population sources for genetic rescue, to characterise temporal genomic erosion and its consequences, and to monitor the outcomes of conservation actions. In this Special Issue, Black et al. (2024) combined genome‐wide data from across the range of the southern brown bandicoot ( *Isoodon obesulus* ) in south‐eastern Australia with simulated introductions to recommend which population may be suitable to restore diversity in a depauperate population. Although the greatest increase in heterozygosity was expected to be reached when using highly differentiated populations as mixing sources, these may represent different subspecies of southern brown bandicoot. The results showed that closely related populations provided a sustained increase to heterozygosity over 50 generations, emphasising the value of simulations when selecting source populations for genetic mixing.

Reid et al. (2025) used multiple types of genomics information to inform and help plan future genetic rescue attempts for the Arkansas darter ( *Etheostoma cragini* ). Genetic structure was pronounced, but there was little evidence of recent inbreeding or accumulation of deleterious variation in the smallest populations. Additional consideration of putatively adaptive variation and structural variation allowed the authors to recommend a series of translocations that should minimize outbreeding depression while maximizing the chances of alleviating inbreeding depression. The authors argued for the use of genetic rescue translocations at earlier stages of population decline; that is, prior to major fitness declines due to inbreeding. This is an important and thought‐provoking consideration for genetic rescue. If we wait until a population has dramatically declined, assisted gene flow‐motivated translocations should have a larger effect on fitness, but inbreeding might re‐accrue quickly if other extrinsic causes of decline have not been addressed. Earlier in the process of population decline might be a better option, but this is likely to be a harder pill to swallow for managers worried about biotic homogenization.

For reintroduced populations, is it better to use one source population or multiple? Halford et al. (2025) explored reintroduced populations of the chequered skipper butterfly ( *Carterocephalus palaemon* ) in England and found that one source population did far better than the other in survival. They examined two source populations in Belgium. Four generations after 66 individuals were released, the effective population size (*N*_e_) was only 33, but most of the source genetic variation was maintained. High relatedness within the introduced populations suggested inbreeding could be a concern in the near future. The authors concluded that one of the source populations dominated the ancestry in the reintroduced populations. The reasons for one source to win out are not entirely clear, but the intriguing possibility of interactions among strains of *Wolbachia* is explored. If a single source is better in this case, the authors' suggestion that translocating more individuals from the same source might be the better management option.

Thavornkanlapachai et al. (2025) also examine mixed‐source population reintroduction, in this case for two island source populations of dibbler ( *Parantechinus apicalis* ), in Australia. Source populations were highly genetically divergent and genetically depauperate. Realized founder population contribution was initially unequal. However, extreme hybrid success led to more equal source population contribution over time. Concurrent examination of mating success in captive animals revealed that higher mating success of younger, heavier animals seemed to explain the initial unequal founder population contribution, but results from captive animals were unable to shed light on the observed high hybrid fitness observed in the wild, reinforcing the need for natural‐based experiments to understand translocation outcomes. A signal of declining genetic variation at the end of the time period examined suggested that genetic monitoring is needed.

Pazhenkova et al. (2025) used comprehensive genetic monitoring and individual‐based genetic‐demographic modelling to assess the long‐term viability of the Dinaric population of Eurasian lynx ( *Lynx lynx* ) post‐translocation, and to formulate effective conservation strategies. This population, which became extinct at the beginning of the 20th century and was re‐established by reintroduction in the 70s, suffers from multiple genetic and fitness issues, is in decline, and has been recently targeted by population reinforcements (synonymous with actions to promote genetic rescue) to prevent re‐extinction. The authors found that recent translocations boosted genetic diversity, but that inbreeding is expected to return to critical levels within 45 years, stressing the importance of additional translocations and effective long‐term genetic management as conservation strategies for genetically impoverished populations.

As with the case of the Eurasian lynx above, conservation translocations for (re)introduction and reinforcement of populations usually involve limited numbers of individuals from constrained sources and typically come with the risk of loss of genetic diversity. Taylor et al. (2024) exemplified the importance of characterization and monitoring of the genetic impacts of conservation translocations focusing on the reintroduction of the Eurasian beaver ( *Castor fiber* ) to Knapdale, Scotland. This translocation occurred without genetic data for the founders. Applying a high‐density SNP panel, the authors showed that initial translocation led to low genetic diversity and high mean kinship, but reinforcement translocations reversed this. Future admixture between the two genetic lineages that now co‐exist at Knapdale is necessary for the full genetic benefits of genetic reinforcement to accrue, which will require future genetic monitoring. The authors use the case to encourage proactive genetic sampling of all founders in such situations.

It is not uncommon for captive populations to have been established during the period of severe declines of wild populations; hence, captive populations may represent important sources of genetic variation for reintroduction into the wild. Al Hikmani et al. (2024) explored whether captive critically endangered Arabian leopards ( *Panthera pardus nimr* ) harbored genetic diversity lost in the wild. This large cat no longer occurs in most of its former range across the Arabian Peninsula, with only 120 individuals remaining in the wild, supplemented by an additional 64 captive leopards. Captive individuals possessed genetic variation not detected in the wild, and outcomes of different genetic rescue scenarios based on introductions of captive leopards were projected using computer modelling. The analyses indicated that to improve the long‐term viability of the wild population, the introduction of only small numbers of captive individuals would likely achieve the optimal trade‐offs among levels and expression of genetic load and genomic erosion.

Bell et al. (2025) used a large genomic dataset to quantify how isolation has shaped genetic diversity and inbreeding in westslope cutthroat trout (*Oncorhynchus lewisi*) in the United States, and to ask whether adaptive divergence in key life‐history traits might complicate genetic rescue decisions. They showed striking heterogeneity across populations, with connected Columbia River basin populations having retained relatively high heterozygosity, whereas several small, isolated populations show extremely low variation and high individual inbreeding. Genome scans for loci associated with life‐history differences among isolated populations revealed only modest evidence for a small number of candidate trait‐associated loci. Overall, the paper argues that while adaptive differentiation should be considered, the severity of inbreeding in the most genetically depauperate populations indicates potential for genetic rescue benefits in fragmented salmonid populations.

In a study of Macquarie perch ( *Macquaria australasica* ), an endangered freshwater fish from Australia, Pavlova et al. (2024) carried out genetic monitoring after a series of translocations. Using genome‐wide SNP data, reconstruction of parent–offspring/full‐sib relationships, and cohort assignment based on body size, they tested whether immigrants reproduced and increased genetic diversity, and evaluated how annual river flow influenced recruitment, dispersal, and inbreeding depression. They found strikingly low levels of admixture, indicating very limited realized gene flow despite substantial releases. Low‐flow years were associated with fewer breeders and lower genetic diversity, and the lowest‐flow cohort showed evidence consistent with inbreeding depression in juvenile growth. The authors conclude that the effectiveness of genetic rescue could be improved by targeting upstream releases and breeding access, while also emphasizing that maintaining adequate flows to enable movement may be essential for both demographic recovery and successful genetic rescue.

Eronen et al. (2024) experimentally hybridized the critically endangered Saimaa landlocked salmon with a nearby anadromous Baltic salmon lineage, then tested how hybridization interacts with a common disease affecting this species, an eye‐fluke infection, to influence juvenile survival under predation. They found that hybrids had substantially lower predation mortality than pure landlocked salmon, consistent with a potential short‐term genetic rescue benefit. However, that advantage was cancelled by parasite infection, which impaired vision (cataracts) and increased susceptibility to predation. This study underscores that the benefits of increasing heterozygosity via hybridization can be strongly context‐dependent—mediated by ecological stressors including disease and predation that may amplify or erase first‐generation fitness gains.

### 2.2 Exploring Past, Present, and Future Conditions for Genetic Rescue

Investigating demographic and adaptive responses to biological invasions and to biotic interactions under climate change can provide insights into the potential for demographic and genetic rescue and the evolutionary resilience of populations. Salamon et al. (2024) combined a lab‐based reciprocal transplant experiment with population genomic analyses of wild samples to examine the interaction between adaptation and demography in the native gastropod *Amnicola limosus* after ~12 years of exposure to an invasive predator, the round goby ( *Neogobius melanostomus* ). They identified patterns of life‐history variation and genetic differentiation consistent with local adaptation to an environmental gradient in the Upper St. Lawrence River, Canada, and to round goby predation. Their results indicate that natural dispersal constrains the potential for demographic and genetic rescue, underscoring the importance of accounting for potentially conflicting effects of local adaptation and gene flow when assessing the resilience of biodiversity threatened by invasive predators.

Baiotto and Guzman (2025) explore the conditions under which evolutionary rescue might be most limited. They simulated both habitat loss and environmental change and explored effects of the mean and variance in environmental conditions. Evolutionary rescue in response to increasing temperatures was limited when there was habitat loss, a reduced range of environmental conditions, and if cooler habitats tend to be lost. These authors urge us to consider the breadth of environmental conditions as part of habitat quality and quantity if we hope for evolutionary rescue to occur.

In a study that used field‐based monitoring, whole genomes spanning the past ~150 years and individual‐based simulations, Cavill et al. (2024) described the temporal genomic erosion of the Seychelles magpie‐robin ( *Copsychus sechellarum* ), a bottlenecked species with high inbreeding and some of the lowest recorded levels of genetic diversity among endangered birds. Despite evidence of recent demographic recovery, extant populations exhibited a marked increase in both genetic and realized load, potentially reflecting an unintended consequence of the species recovery program through the relaxation of purifying selection. Simulated scenarios were used to explore how genetic rescue could alter the future trajectory of the species' genomic erosion, setting the stage for forward‐looking strategies to address future genetic and conservation challenges.

Encinas‐Viso et al. (2024) leverage individual‐based spatially explicit simulation models to dissect the relative importance of demographic rescue, genetic rescue, and habitat restoration for stabilizing small and isolated plant populations under rapid climate change. This research focuses specifically on obligately outcrossing plant species, which cannot resort to self‐pollination if abundance and mate availability decline. Rapid climate change in concert with habitat fragmentation and loss increases the vulnerability of these species to decline and ultimately extinction. The simulation model clearly reveals that all three approaches can be viable management options whether alone or in concert. The introduction of novel genetic variation leading to genetic rescue is highly effective for increasing plant fitness and population persistence. Additionally, the simple influx of large numbers of individuals—even without augmenting genetic variation—can forestall extinction in very small populations. Finally, the combination of habitat restoration and genetic rescue appears to be the best strategy for promoting population persistence. This work highlights the importance of implementing multiple management options simultaneously for the preservation of biodiversity under rapid global change.

In a study of biotic interactions, Ge et al. (2024) used ladybirds and aphids to develop an eco‐evolutionary model that explores evolutionary rescue in a predator–prey system under climate change. Geographic variation in thermal performance evolution and evolutionary rescue was detected in the predator but not in the agricultural pest, indicating that interacting species respond differently to the effects of seasonality and warming through ecological versus eco‐evolutionary mechanisms. By advancing the application of species distribution models (SDMs) in agricultural systems, their work provides causal insight into how ecological and evolutionary dynamics interact to drive the evolutionary rescue of a predator responding to a crop pest.

Assisted gene flow can be an ethical and effective strategy for conserving genetic diversity and reducing the risk of local extinction. This conservation approach moves propagules to new populations within the existing range of a species, thus eliminating concerns about moving species beyond their current range edge. Through assisted gene flow, conservationists can introduce alleles adapted to hot and dry climates into populations in historically cooler locations that are now facing increased temperatures and aridification. Sacristán‐Bajo et al. (2025) evaluated assisted gene flow in the annual legume *Lupinus angustifolius* in Spain by crossing southern and northern accessions. The assisted gene flow lines flowered significantly earlier and produced heavier seeds than northern accessions. Furthermore, genomic data detected 36 outlier markers that differed across the control and gene flow lines; these markers are associated with flowering phenology and other traits that differed between the lines. Future studies can assess whether assisted gene flow also reduces the risk of population decline. This approach may not be practical for all species, but it should be considered for ecologically or culturally important species.

## 3 Emerging Tools, Perspectives, and Paradigms for Biodiversity Conservation

It is becoming increasingly clear that structural variants (SVs: insertions, deletions, duplications and inversions) in genomes are important components of species' genomic diversity and relevant to fitness and adaptation, although data remain scant (Schneller et al. 2025). In this Special Issue, Smeds et al. (2024) contributed significantly to the current knowledge of structural genomic variation in endangered species by analysing whole‐genome sequences from hundreds of inbred Scandinavian wolves ( *Canis lupus* ) and from neighbouring populations in Finland and Russia, detecting > 26,000 high‐confidence SVs, most of which were < 1 kb, with deletions of 190 bp being particularly common, corresponding to insertions of SINE/tRNA‐Lys elements. It was inferred that SVs in protein‐coding regions were generally under purifying selection. The realized genetic load of these SVs increased with inbreeding levels in the Scandinavian population, which was counteracted by immigration acting to restore diversity at loci otherwise fixed for derived SVs.

As biodiversity loss accelerates, opportunities for restorative actions diminish and so conservationists are increasingly considering novel tools for fighting extinction, including making use of biobanked cells. Assessment of the genomic health of such resources is necessary to understand their potential for restoring viable populations. Wilder et al. (2024) used forward‐simulations of a future restored population of the effectively extinct northern white rhinoceros ( *Ceratotherium simum cottoni* ). This was informed by empirical genomic variation of available cryopreserved founders. The banked genomes had higher genetic diversity and less realized genetic load but more masked genetic load than the sister subspecies southern white rhino (*C. s. simum*), which has been able to recover from a severe bottleneck. The authors estimated the impact of this genetic load on the fitness of a northern white rhinoceros population restored from biobanked cells, comparing the outcomes for single versus repeated introductions of the same founders over generations. The results suggested that the ability to introduce biobanked founder cells repeatedly could help restore genetic variation from even a limited founder pool, which may contribute to the range of strategies to counter the extinction crisis.

In a perspective that explored the development and application of computational models to inform genetic rescue, Beaman et al. (2025) provided a practical roadmap for building genetically explicit, individual‐based demo‐genetic simulations. Their guide incorporated key mechanisms (e.g., partially recessive deleterious mutations and demographic rates whose variance increases as abundance declines), and they also compared the capabilities of five open‐source simulation programs. Using a heuristic example, they showed that simulated rescue can delay extinction in small populations under demo‐genetic feedback and then illustrated how published genetic data from threatened Australian marsupials can be used to parameterize, calibrate, and validate models. Finally, they recommend using simulations to rank alternative translocation strategies (considering parameters such as size, frequency, and source choice) by sensitivity of predicted extinction probability to those parameters—explicitly framing their approach as a decision‐support tool for genetic rescue.

Conservationists often rely upon accepted taxonomic names for drafting policy and implementing management actions. Clavero et al. (2024) consider the ramifications of defining species too narrowly for conservation of imperilled populations, illustrating this point through a case study of the western capercaillie ( *Tetrao urogallus* , Phasianidae), the largest species of grouse currently in existence. This species has an incredibly broad geographic distribution, from Spain to Russia, but many populations in the western portion of the range are small and isolated. These threatened populations have been divided into at least 13 subspecies, despite lack of support for this splitting from phylogenetic studies based on mitochondrial DNA data. Clavero et al. (2024) highlight that the legal listing of the highly inbred population of *T. urogallus* in the Cantabrian Mountains of Spain as a subspecies conflicts with these recent molecular data. This Cantabrian capercaillie population is facing extinction, even though genetic rescue from other populations of this species could reduce inbreeding depressions and augment population size. Thus, taxonomic inflation, or the splitting of species into smaller and smaller units, could inhibit conservation actions that may preserve declining populations. This study offers a very pragmatic perspective on taxonomy in a time of rapid environmental change when populations face diminishing genetic diversity and novel environmental conditions.

The essential metabolic genes encoded by the mitochondrial genome represent a key component of the genetic variation underpinning organisms' ecophysiology and can be key drivers facilitating climatic adaptation. Because large numbers of species are unlikely to shift their ranges or adapt sufficiently quickly to keep pace with climate change, there are calls for interventions intended to augment adaptive genetic variation and thus accelerate evolutionary change. In the case of the mitochondrial genome, such approaches would need to take into account that mitochondrially encoded genes must interact biochemically and evolutionarily with nuclear encoded genes with mitochondrial functions. These coadapted mitonuclear genes form some of the important reproductive barriers between species. Iverson (2024) contended that conservation decision‐making needs to consider the relevance of mitonuclear genetic variability in assisted evolution. Iverson proposed a novel technique, Conservation Mitonuclear Replacement (CmNR), as an approach to replace the mitochondrial genome and key interacting nuclear loci of a threatened species with those from sources more suited to the climates in which species are constrained to occur. Iverson considers the most plausible pathway to CmNR combines CRISPR‐based nuclear gene editing with mitochondrial replacement and assisted reproductive technologies, preserving much of an organism's phenotype, with the goal that populations could persist in the wild when no other suitable conservation options exist.

## 4 Conclusions

The ability to leverage genetic rescue through improved management actions and monitoring is expected to increase as a result of recent advances in population, ecological and conservation genomics. This includes access to, or the capacity to generate, cost‐effective large‐scale omics datasets for virtually any wild or captive species, and the ability to integrate these data analytically with environmental, experimental, historical, and demographic data (Zamudio 2023; von der Heyden et al. 2025). This Special Issue presents a diverse range of applications and perspectives on genetic and evolutionary rescue as tools for conserving threatened biodiversity. The assembled contributions outline recent advances in the field and their implications for conservation policy and practice. Collectively, these studies demonstrate how insights from genomics and evolutionary biology can inform evidence‐based decision‐making, risk assessment, and the design of effective management interventions, including crucial monitoring across a variety of ecological and socio‐environmental contexts. By highlighting the opportunities and limitations associated with genetic and evolutionary rescue, this Special Issue supports the integration of these approaches into conservation planning and policy frameworks. In doing so, it emphasizes their potential to enhance the resilience, adaptive capacity, and long‐term persistence of biodiversity in the face of accelerating environmental change.

## Conflicts of Interest

The authors declare no conflicts of interest.

## Data Availability

Data sharing not applicable to this article as no datasets were generated or analysed during the current study.
